# Adenomyosis as a prognostic factor in ovarian cancer: a retrospective study

**DOI:** 10.1007/s00404-025-08023-5

**Published:** 2025-04-16

**Authors:** Gozde Sahin, Isil Turan Bakirci, Isik Sozen, Sinem Ozsahin Kilic, Selim Afsar, Nilufer Cetinkaya Kocadal, Ipek Geyikoglu

**Affiliations:** 1 Division of Gynaecologic Oncology, Department of Obstetrics and Gynecology, Basaksehir Cam and Sakura City Hospital, Istanbul, Turkey; 2 Division of Perinatology, Department of Obstetrics and Gynecology, Basaksehir Cam and Sakura City Hospital, Istanbul, Turkey; 3 Division of Gynaecologic Oncology, Department of Obstetrics and Gynecology, Balikesir University, School of Medicine, Balikesir, Turkey; 4 Department of Obstetrics and Gynecology, Basaksehir Cam and Sakura City Hospital, Istanbul, Turkey

**Keywords:** Ovarian cancer, Adenomyosis, Prognosis, Survival, Histological subtypes

## Abstract

**Background:**

Adenomyosis is a gynecological condition that frequently coexists with gynecological malignancies and has been shown to influence disease outcomes. However, its impact on ovarian cancer prognosis remains unclear. This study aimed to investigate the relationship between adenomyosis and clinicopathological and prognostic features in ovarian cancer patients.

**Methods:**

We retrospectively analyzed 226 patients with ovarian cancer who underwent surgery between 2020 and 2023. The patients were divided into two groups based on the presence (n = 114) or absence (n = 112) of adenomyosis, confirmed by histopathological examination. Clinicopathological characteristics, including histological subtypes, disease-free survival (DFS), and overall survival (OS) were compared between the groups with a median follow-up of 36 months.

**Results:**

Patients with adenomyosis demonstrated more favorable characteristics, including early stage disease (54.3% vs 39.2%, p = 0.048), lower-grade tumors (55.2% vs 31.2%, p = 0.049), and smaller tumor sizes (39.4% vs 26.7%, p = 0.043). Adenomyosis was significantly associated with endometrioid subtype (OR = 2.89, p = 0.043) and negatively associated with serous carcinoma (OR = 0.39, p = 0.034). Three-year DFS was significantly better in the adenomyosis group (79.2% vs 73.9%, p = 0.01), particularly in high-grade tumors (80% vs 58%, p < 0.05). No significant difference was observed in overall OS (73.3% vs 73.1%, p = 0.14), although high-grade tumors with adenomyosis showed improved OS (71% vs 57%, p < 0.05).

**Conclusion:**

The presence of adenomyosis in patients with ovarian cancer was associated with favorable clinicopathological features, particularly endometrioid histology and low-grade tumors, and improved survival in high-grade tumors. These findings suggest a potential biological interaction between adenomyosis and ovarian cancer that warrants further investigation for personalized treatment approaches.

## Introduction

Ovarian cancer is one of the leading causes of cancer-related deaths among women, with an overall five-year survival rate of less than 55% [1–4]. The high mortality rate associated with ovarian cancer is largely due to late-stage diagnoses, as early symptoms are often nonspecific and there are currently no reliable screening methods for early detection. Prognostic factors that significantly affect survival include stage at diagnosis, histological subtype, tumor grade, and patient performance status. Specifically, advanced stage and high-grade histology are associated with poor prognoses. Despite recent advancements in treatment, including targeted therapies and PARP inhibitors, survival rates remain disappointing, particularly in patients diagnosed at an advanced stage [4].

Adenomyosis is characterized by the presence of endometrial tissue within the myometrium and affects approximately 20–30% of women, particularly those in their late reproductive years or menopause [5, 6]. Adenomyosis frequently coexists with gynecological malignancies and its presence has been shown to influence disease outcomes in various gynecological cancers [7]. Although generally considered a benign condition, malignant transformation in adenomyosis is rare, occurring in only approximately 1% of cases, primarily in older individuals [8–10]. The pathogenesis of malignant transformation is complex and involves multiple mechanisms, including hormonal factors, genetic predisposition, growth factors, inflammation, immune system dysregulation, environmental factors, and oxidative stress [8]. Similar to other gynecological malignancies, local hyperestrogenic effects contribute to the development of adenomyosis, suggesting potential interactions between adenomyosis and various gynecological cancers via shared hormonal pathways [7]. Adenomyosis is more frequently associated with endometrioid carcinoma, although rare cases of clear cell carcinoma have also been reported [8].

Studies suggest a possible association between adenomyosis and ovarian cancer risk. Shen et al. (2020) found a 2.3-fold increase in ovarian cancer risk in women with adenomyosis [2]. Hermens et al. (2021) reported a significantly higher incidence of endometrioid ovarian cancer in patients with histologically proven adenomyosis (adjusted IRR = 2.51). Diagnostic challenges in identifying malignant transformation, particularly in clear cell carcinoma, may delay diagnosis and worsen outcomes [10].

However, the relationship between adenomyosis and cancer prognosis remains controversial. While some studies suggest that adenomyosis may be associated with greater myometrial invasion, others indicate lower aggressiveness and fewer recurrences in cases where adenomyosis is present. The most significant risk factor for malignant transformation appears to be prolonged exposure to estrogen, with hormone replacement therapy being particularly notable. Ectopic endometrial tissue can produce estrogen through autocrine and paracrine effects, potentially explaining the persistence and recurrence of tumors, even in postmenopausal women [8].

Despite these emerging findings, previous studies have either not addressed the prognosis of ovarian cancer with adenomyosis or yielded conflicting results regarding its impact on survival outcomes. Some studies suggest that adenomyosis may have a protective effect by modulating the tumor microenvironment, whereas others indicate no significant impact or even a potential for increased invasiveness.

This study aimed to address this gap by comprehensively investigating the relationship between adenomyosis and ovarian cancer prognosis, particularly focusing on its association with clinicopathological characteristics, histological subtypes, survival outcomes, and potential prognostic implications.

## Materials and methods

This retrospective study included patients with ovarian cancer who underwent surgery at Basaksehir Cam and Sakura City Hospital in Istanbul, Turkey between July 2020 and October 2023. During this period, 255 women underwent surgery for ovarian cancer. Patients who received neoadjuvant chemotherapy (n = 12), had synchronous malignancy (n = 4), incomplete medical records (n = 8), or experienced local or distant metastatic disease within 3 months after primary surgery (n = 5) were excluded. A total of 226 patients were included in the final analysis with a median follow-up period of 36 months. Of these, 114 (50.4%) patients had adenomyosis, whereas the remaining 112 (49.6%) did not. The sample size was determined based on the number of cases available during the study period.

All surgeries were performed by gynecological oncologists, and included comprehensive staging with total abdominal hysterectomy, bilateral salpingo-oophorectomy, omentectomy, and systematic pelvic and para-aortic lymph node dissection. Intraoperative frozen section consultations were conducted following hysterectomy. Tumor size was measured in centimeters by pathologists. Adenomyosis was confirmed exclusively by histopathological examination and defined as the presence of endometrial glands and stroma at a depth of 2.5 mm or more from the endomyometrial junction. Pathological analysis was conducted by gynecological pathologists, and tumors were graded according to the International Federation of Gynecology and Obstetrics criteria. Lymphovascular space invasion (LVSI) was defined as the presence of tumor cells in the lymphatic or capillary lumen.

Patient data, including age, preoperative CA-125 levels (cut-off value: 35 U/mL), histological subtype (serous, endometrioid, clear cell, and mucinous), tumor grade (categorized as low-grade or high-grade for analysis purposes, with grade 1 tumors classified as low-grade and grade 2–3 tumors as high-grade), tumor stage, tumor size, estrogen receptor (ER) status, progesterone receptor (PR) status, LVSI, peritoneal cytology, appendix involvement, omentum involvement, pelvic and para-aortic lymph node involvement, distant metastasis, recurrence, and follow-up data, were extracted from the institutional database. ER and PR statuses were evaluated using immunohistochemistry, with positive staining defined as > 1% of the cells showing nuclear staining. Patients were categorized into the adenomyosis and non-adenomyosis groups for comparative analysis.

The primary endpoints were overall survival (OS), defined as the time from surgery to death from any cause, and disease-free survival (DFS), defined as the time from surgery to the first documented recurrence.

### Statistical analysis

Statistical analyses were performed using the IBM SPSS Statistics for Windows (version 21.0; IBM Corp., Armonk, NY, USA). Missing data were handled by imputation, where feasible, based on the most common values or means within subgroups. Multicollinearity was assessed for variables included in the Cox regression analysis. Univariate and multivariate analyses were conducted to examine factors associated with OS and DFS. The variables included in these analyses were age, CA-125 level, tumor stage, tumor grade, tumor size, ER status, PR status, LVSI, and lymph node involvement. The normality of the data distribution was evaluated using the Kolmogorov–Smirnov and Shapiro–Wilk tests. Continuous variables were expressed as median (range) and compared using the independent-samples t-test or Mann–Whitney U test, as appropriate. Categorical variables are presented as counts and percentages and were analyzed using the chi-squared test or Fisher’s exact test. Survival curves were constructed using the Kaplan–Meier method, and the log-rank test was used for comparison. The Cox regression model was used to identify the predictors of DFS and OS. The logistic regression analysis was performed to evaluate the association between adenomyosis, histological subtypes and tumor grade. Statistical significance was set at p < 0.05.

The study was approved by the Ethics Committee of Basaksehir Cam and Sakura City Hospital (approval number: 24.08.2022/260).

## Results

Among 255 women who underwent surgery for ovarian cancer between July 2020 and October 2023, 226 met the inclusion criteria after excluding those who received neoadjuvant chemotherapy (n = 12), had synchronous malignancy (n = 4), incomplete medical records (n = 8), or early metastatic disease (n = 5). Adenomyosis was observed in 114 (50.4%) patients. The median age at diagnosis was 55 years (range: 28–83), with the adenomyosis and non-adenomyosis groups having median ages of 53 and 57 years, respectively (p = 0.12) (Table 1).

**Table 1:** Clinicopathological characteristics of patients stratified by adenomyosis status

Subject	Adenomyosis n = 114 (50.4%)	Non-adenomyosis n = 112 (49.6%)	*p* value
*Age (years), median (range)*	53 (28–82)	57 (30–83)	0.12
*CA 125 (IU/L)*			0.47
< 35	28 (24.5)	24 (21.4)	
≥ 35	86 (75.5)	88 (78.6)	
*Estrogen receptor*			0.45
No	41 (35.9)	35 (31.2)	
Yes	73 (64.1)	77 (68.8)	
*Progesterone receptor*			0.44
No	56 (49.1)	50 (44.6)	
Yes	58 (50.9)	62 (55.4)	
*Histological subtype*			< 0.001
Clear cell carcinoma	16 (14.04)	9 (8.04)	
Endometrioid carcinoma	35 (30.70)	9 (8.04)	
Mucinous carcinoma	4 (3.51)	8 (7.14)	
Serous carcinoma	59 (51.75)	86 (76.79)	
*Grade classification*			0.002
Low-grade	35 (30.70)	15 (13.39)	
High-grade	79 (69.30)	97 (86.61)	
*Stage*			0.048
I	52 (45.6)	39 (34.8)	
II	10 (8.7)	5 (4.4)	
III	32 (28.1)	46 (41.1)	
IV	20 (17.6)	22 (19.7)	
*Tumor size (cm)*			0.043
≤ 4	45 (39.4)	30 (26.7)	
> 4	69 (60.6)	82 (73.3)	
*Peritoneal cytology*			0.23
Negative	69 (60.5)	59 (52.6)	
Positive	45 (39.5)	53 (47.4)	
LVSI			0.37
No	58 (50.8)	46 (41.1)	
Yes	56 (49.2)	66 (58.9)	
*Appendix involvement*			0.28
No	95 (83.3)	87 (77.6)	
Yes	19 (16.7)	25 (22.4)	
*Omentum involvement*			0.23
No	70 (61.4)	60 (53.5)	
Yes	44 (38.6)	52 (46.5)	
*Pelvic lymph node involvement*			0.39
No	88 (77.1)	79 (70.5)	
Yes	26 (22.9)	33 (29.5)	
*Para-aortic lymph node involvement*			0.45
No	89 (78.1)	79 (70.5)	
Yes	25 (21.9)	33 (29.5)	
*Distant metastasis*			0.43
No	77 (67.5)	66 (58.9)	
Yes	37 (32.5)	46 (41.1)	

CA 125, cancer antigen 125; LVSI, lymphovascular space invasion

Patients with adenomyosis demonstrated significantly favorable characteristics, including early stage disease (54.3% vs. 39.2% for stages I–II, p = 0.048), lower-grade tumors (30.7% vs. 13.3% p = 0.002), and smaller tumor size (39.4% vs. 26.7% for ≤ 4 cm, p = 0.043) (Table 1). Adenomyosis was significantly associated with endometrioid subtype (35 vs. 9, OR = 2.89, p = 0.043) and negatively associated with serous carcinoma (59 vs. 86, OR = 0.39, p = 0.034). No significant association was found between adenomyosis and clear cell carcinoma (16 vs. 9, OR = 1.77, p = 0.167) or mucinous carcinoma (4 vs. 8, OR = 0.28, p = 0.087) (Table 2).

**Table 2:** Logistic regression analysis of histologic subtype and grade according to the presence of adenomyosis

*Histological subtype*	OR	95%CI	p
Clear cell carcinoma	1.77	0.78–4.02	0.167
Endometrioid carcinoma	2.89	1.13–6.55	0.043
Mucinous carcinoma	0.28	0.07–1.20	0.087
Serous carcinoma	0.39	0.16–0.93	0.034
*Grade classification*			
Low grade	3.06	1.45–6.45	< 0.05
High grade	0.34	0.17–0.68	< 0.05

OR, odds ratio; CI, confidence interval

According to logistic regression analysis, patients with adenomyosis were significantly more likely to have low-grade tumors (OR = 3.06, 95% CI: 1.45–6.45, p < 0.05) and less likely to have high-grade tumors (OR = 0.34, 95% CI: 0.17–0.68, p < 0.05) (Table 2).

**Fig. 1:**
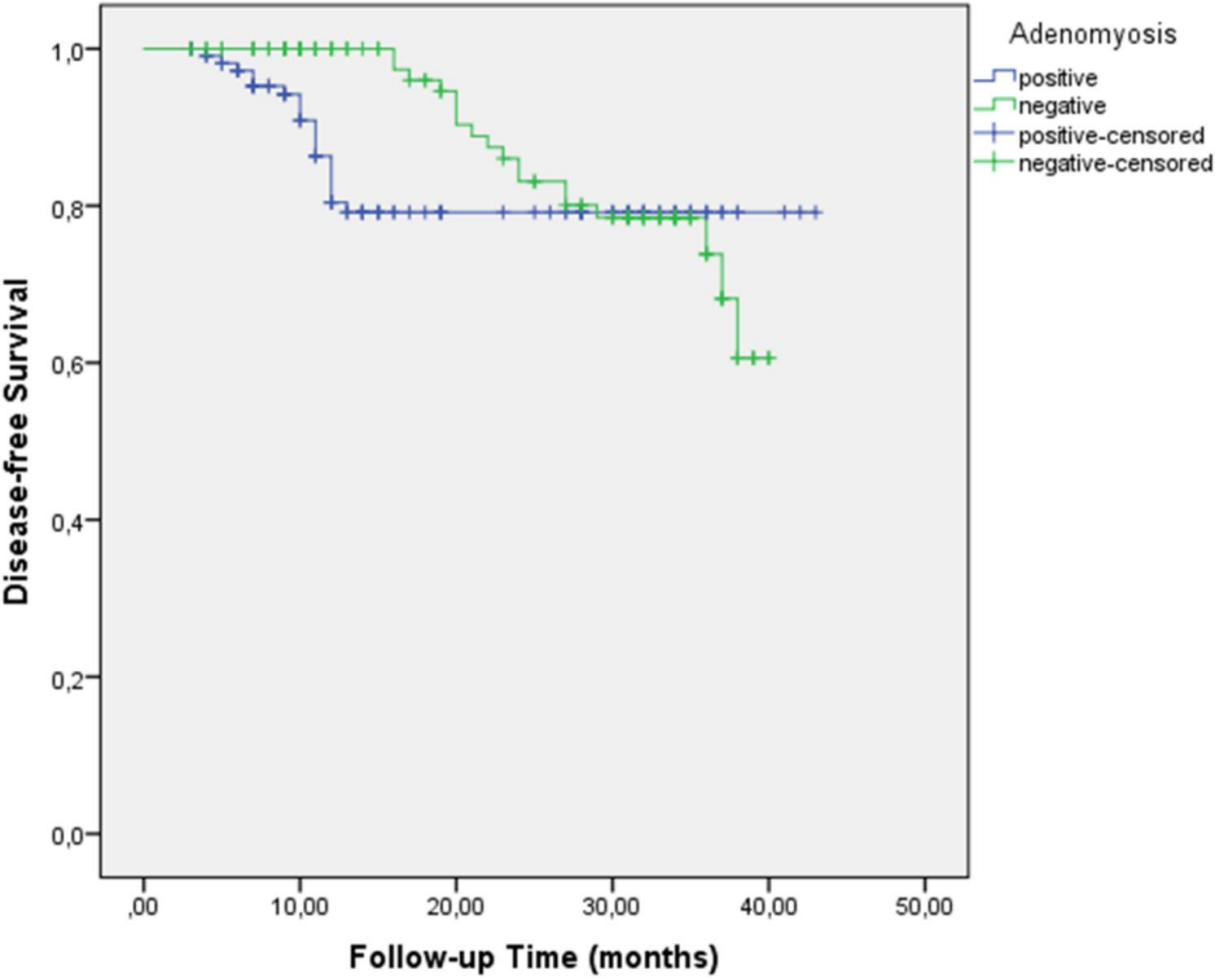
Kaplan–Meier curve for disease-free survival in ovarian cancer patients with and without adenomyosis

**Fig. 2:**
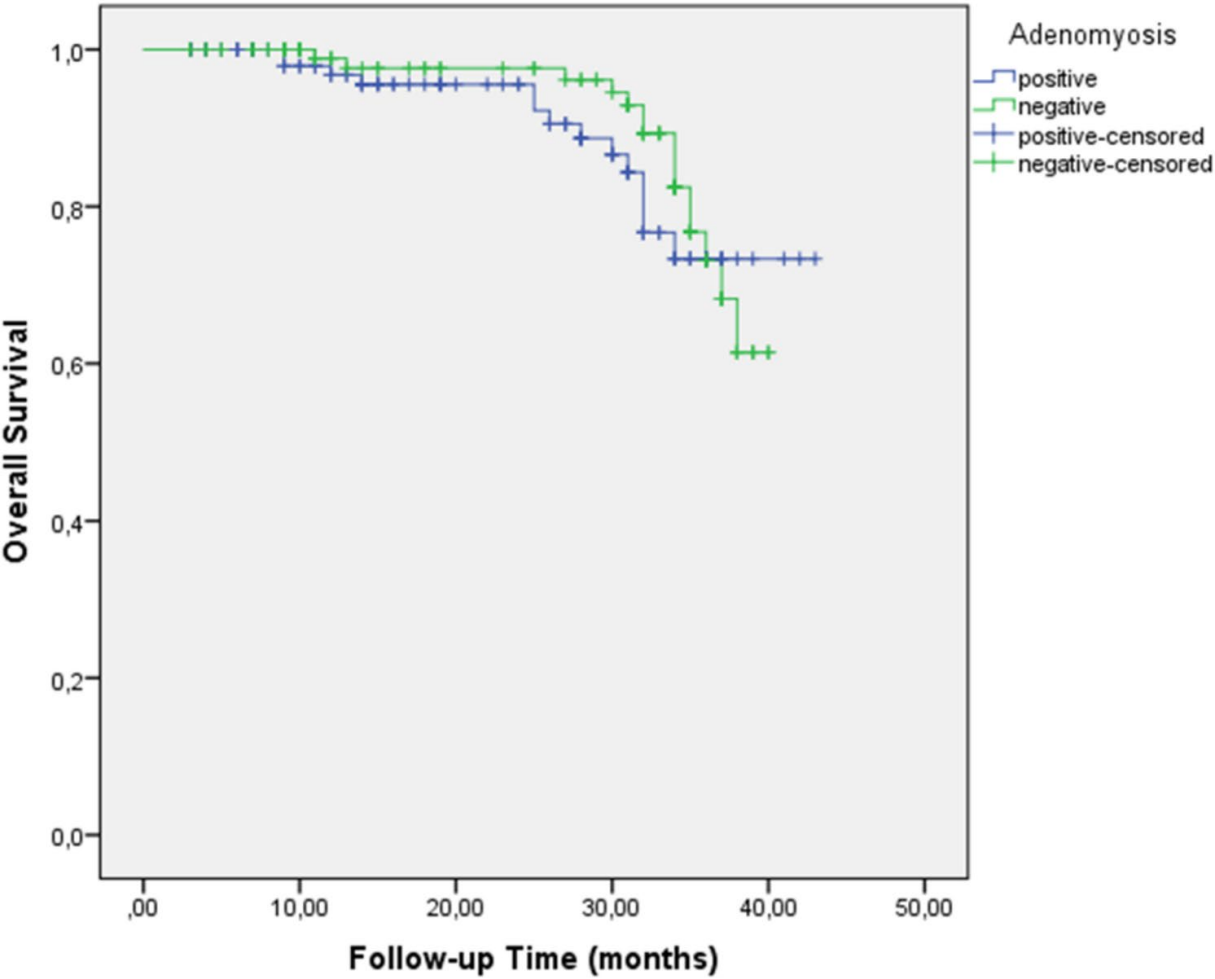
Kaplan–Meier curve for overall survival in ovarian cancer patients with and without adenomyosis

With a median follow-up of 36 months, patients with adenomyosis had significantly improved 3-year disease-free survival (DFS) compared to those without adenomyosis (79.2% vs. 73.9%, p = 0.01) (Fig. 1 for Kaplan–Meier survival curves with patient numbers at risk). The number of patients at risk at 0, 12, 24, and 36 months was 114, 98, 85, and 72, respectively, for the adenomyosis group and 112, 92, 76, and 61, respectively, for the non-adenomyosis group. However, no significant difference was observed in the 3-year overall survival (OS) between the groups (73.3% vs. 73.1%, p = 0.14) (Fig. 2, Table 3).

**Table 3:** Three-year disease-free and overall survival outcomes based on adenomyosis status

	Adenomyosis	Non-adenomyosis	*p* value
3-year DFS (%)	79.2	73.9	0.01
3-year OS (%)	73.3	73.1	0.14
*Low-grade tumors*			
3-year DFS (%)	74	75	0.98
3-year OS (%)	81	84	0.93
*High-grade tumors*			
3-year DFS (%)	80	58	< 0.05
3-year OS (%)	71	57	< 0.05

OS, overall survival; DFS, disease-free survival

Subgroup analysis revealed that adenomyosis was not associated with significant differences in DFS or OS in low-grade tumors (DFS: 74% vs. 75%, p = 0.98; OS: 81% vs. 84%, p = 0.93). However, in high-grade tumors, adenomyosis was associated with significantly improved DFS (80% vs. 58%, p < 0.05) and OS (71% vs. 57%, p < 0.05).

In univariate analysis, factors associated with improved DFS included low-grade tumor (p = 0.02), early stage disease (p = 0.007), negative LVSI (p = 0.04), negative peritoneal cytology (p = 0.01), and absence of lymph node metastasis (p < 0.001) (Table 4). Multivariate analysis revealed that the absence of para-aortic lymph node metastasis emerged as an independent predictor of improved DFS (hazard ratio [HR] = 6.35, 95% CI: 2.39–16.88, p = 0.001).

**Table 4:** Univariate and multivariate predictors of disease-free survival

Variables	Event, n (%)	Univariate analysis	Multivariate analysis			
		*p* value	HR	95% CI		*p* value
*Age (years)*		0.27	0.57	0.26–1.26		0.16
< 60	27/146 (18.4)					
≥ 60	10/80 (12.5)					
*Adenomyosis*		0.35	0.61	0.29–1.26		0.18
No	18/112 (16.1)					
Yes	19/114 (16.6)					
*CA 125 (IU/L)*		0.27	0.96	0.60–1.52		0.86
< 35	5/51 (9.8)					
≥ 35	32/174 (18.3)					
*Estrogen receptor*		0.21	0.53	0.17–1.61		0.26
No	10/76 (13.1)					
Yes	27/150 (18)					
*Progesterone receptor*		0.86	1.68	0.68–4.16		0.25
No	17/105 (16.1)					
Yes	20/121 (16.5)					
*Grade classification*		0.02	1.34	0.58–3.09		0.48
Low-grade	3/50 (6.0)					
High-grade	34/176 (19.3)					
*Stage*		0.007	1.19		0.71–1.99	0.49
I–II	8/100 (8)					
III–IV	29/126 (17.1)					
*Tumor size (cm)*		0.50	1.96		0.79–4.82	0.14
≤ 4	8/57 (14.1)					
> 4	29/169 (17.1)					
*Peritoneal cytology*		0.01	1.96		0.79–4.82	0.14
Negative	15/128 (11.7)					
Positive	22/98 (22.4)					
*LVSI*		0.04	0.60		0.22–1.66	0.33
No	11/98 (11.2)					
Yes	26/128 (20.3)					
*Appendix involvement*		0.47	0.55		0.21–1.42	0.22
No	27/182 (14.8)					
Yes	10/44 (22.7)					
*Omentum involvement*		0.07	0.59		0.19–1.78	0.35
No	16/130 (12.3)					
Yes	21/96 (21.8)					
*Pelvic lymph node involvement*		0.002	1.37		0.50–3.78	0.53
No	18/161 (11.1)					
Yes	19/65 (29.2)					
*Para-aortic lymph node involvement*		< 0.001	6.35		2.39–16.88	< 0.001
No	14/159 (8.8)					
Yes	23/67 (34.3)					

CA 125, cancer antigen 125; CI, confidence interval; HR, hazard ratio; LVSI, lymphovascular space invasion

Univariate analysis identified early stage disease (p = 0.04), negative LVSI (p = 0.01), absence of para-aortic lymph node metastasis (p = 0.03), and absence of distant metastasis (p = 0.006) as favorable prognostic factors (Table 5). Independent predictors of improved OS in multivariate analysis included negative LVSI (hazard ratio [HR] = 3.85, 95% CI: 1.19–12.50, p = 0.02), absence of para-aortic lymph node metastasis (HR = 3.22, 95% CI: 1.02–10.12, p = 0.04), and absence of distant metastasis (HR = 7.06, 95% CI: 1.35–36.73, p = 0.02).

**Table 5:** Predictors of overall survival: univariate and multivariate analyses

Variables	Event, n (%)	Univariate analysis	Multivariate analysis				
		*p* value	HR	95% CI		*p* value	
*Age (years)*		0.78	0.92	0.39–2.13		0.85	
< 60	19/146 (13.1)						
≥ 60	10/80 (12.5)						
*Adenomyosis*		0.50	0.56	0.24–1.31		0.18	
No	15/112 (13.3)						
Yes	14/114 (12.2)						
*CA 125 (IU/L)*		0.38	0.80	0.23–2.71		0.72	
< 35	4/51 (7.8)						
≥ 35	25/174 (14.3)						
*Estrogen receptor*		0.35	0.80	0.23–2.71		0.72	
No	9/76 (11.8)						
Yes	20/150 (13.3)						
*Progesterone receptor*		0.19	0.46		0.14–1.50	0.20	
No	11/105 (10.4)						
Yes	18/121 (14.8)						
*Grade classification*		0.15	0.84		0.31–2.27	0.73	
Low-grade	3/50 (6.0)						
High-grade	26/176 (14.7)						
*Stage*		0.04	0.77		0.40–1.46		0.42
I–II	5/100 (5)						
III–IV	24/126 (19.1)						
*Tumor size (cm)*		0.80	0.84		0.32–2.19		0.73
≤ 4	8/57 (14.1)						
> 4	21/169 (12.4)						
*Peritoneal cytology*		0.28	0.94		0.36–2.42		0.89
Negative	14/128 (10.9)						
Positive	15/98 (15.3)						
*LVSI*		0.01	3.85		1.19–12.50		0.02
No	6/98 (6.1)						
Yes	23/128 (17.9)						
*Appendix involvement*		0.06	1.79		0.65–4.92		0.25
No	17/182 (9.3)						
Yes	12/44 (27.2)						
*Omentum involvement*		0.08	0.62		0.18–2.11		0.44
No	11/130 (8.4)						
Yes	18/96 (18.7)						
*Pelvic lymph node involvement*		0.55	0.44		0.35–1.31		0.06
No	18/161 (11.1)						
Yes	11/65 (16.9)						
*Para-aortic lymph node involvement*		0.03	3.22		1.02–10.12		0.04
No	14/159 (8.8)						
Yes	15/67 (22.3)						
*Distant metastasis*		0.006	7.06		1.35–36.73		0.02
No	6/130 (4.6)						
Yes	23/96 (23.9)						

CA 125, cancer antigen 125; CI, confidence interval; HR, hazard ratio; LVSI, lymphovascular space invasion

## Discussion

This study demonstrated that ovarian cancer patients with adenomyosis tend to have more favorable clinicopathological features, such as earlier-stage disease, lower tumor grade, and smaller tumor size, than those without adenomyosis. Our analysis revealed significant associations between adenomyosis and specific histological subtypes, with a notable predilection for endometrioid carcinoma (odds ratio [OR] = 2.89, p = 0.043) and a negative association with serous carcinoma (OR = 0.39, p = 0.034). Despite the improved disease-free survival (DFS) observed in patients with adenomyosis, particularly in those with high-grade tumors, our multivariate analysis did not establish adenomyosis as an independent prognostic factor for survival.

Previous studies have yielded conflicting results regarding the prognostic implications of adenomyosis in gynecological malignancies. Yilmaz et al. [7] reported that adenomyosis could influence disease outcomes in endometrial cancer, whereas other studies have suggested varying effects across different gynecological cancers. Our research specifically addresses this knowledge gap by comprehensively examining the relationship between adenomyosis and ovarian cancer outcomes, a question that remains underexplored in the current literature.

The association between benign gynecological conditions such as adenomyosis and ovarian cancer may be attributed to shared hormonal and molecular mechanisms. Adenomyosis, characterized by the presence of endometrial tissue within the myometrium, involves complex pathways that include sex steroid hormone receptors, inflammatory molecules, extracellular matrix enzymes, growth factors, and neuroangiogenic factors [11]. These shared molecular mechanisms with endometriosis, particularly the role of estrogen, may contribute to the development of ovarian cancer, especially in specific subtypes, such as endometrioid and clear cell carcinomas [12].

Our findings strongly support this mechanistic relationship, as we observed a significant association between adenomyosis and endometrioid carcinoma, whereas serous carcinoma was negatively associated with adenomyosis. This pattern aligns with established knowledge about endometriosis-associated ovarian cancers, which predominantly include endometrioid and clear cell subtypes, suggesting similar pathogenic mechanisms between adenomyosis and endometriosis in relation to ovarian carcinogenesis. The prevalence of endometrioid histology in adenomyosis-associated ovarian cancers may be explained by the shared origin of endometrial-type tissue and common molecular alterations, including ARID1A mutations and PI3K/AKT pathway activation, which are frequently observed in both endometriosis-associated and adenomyosis-associated malignancies [13, 14].

Unlike the well-established relationship between endometriosis and ovarian cancer, that between adenomyosis and ovarian cancer remains unclear. These contradictory findings across studies may stem from methodological differences, varying diagnostic criteria for adenomyosis (clinical vs. histopathological), and heterogeneous patient populations. Our study uniquely contributes to this discussion by using a strict histopathological confirmation of adenomyosis, which enhances the reliability of our findings.

Similar to endometriosis, adenomyosis has been hypothesized to increase the risk of ovarian cancer. Recent studies have reported varying degrees of increased risk in patients with adenomyosis. For instance, Shen et al. reported a 2.3-fold increase in ovarian cancer risk among women with adenomyosis, and Kok et al. found a hazard ratio of 5.5 for ovarian cancer in women with clinically diagnosed adenomyosis [15, 16]. Our findings align with these studies, indicating an increased frequency of adenomyosis among patients with ovarian cancer, although a clear causal relationship is yet to be established.

Our findings indicate that ovarian cancer patients with adenomyosis presented with more favorable clinicopathological characteristics, such as early stage disease (54.3% vs. 39.2% for stages I-II, p = 0.048), lower tumor grade (30.7% vs. 13.3% p = 0.002), and smaller tumor size (39.4% vs. 26.7% for ≤ 4 cm, p = 0.043). In survival analysis, adenomyosis was associated with improved disease-free survival (DFS) (3-year DFS: 79.2% vs. 73.9%, p = 0.01), although it was not an independent predictor in the multivariate analysis. The absence of adenomyosis as an independent prognostic factor likely reflects the stronger influence of established prognostic factors, such as stage, grade, and lymph node involvement, which may mediate or overshadow the effects of adenomyosis on survival outcomes.

One of the most intriguing findings of our study was the differential impact of adenomyosis on survival based on tumor grade. Although adenomyosis showed no significant effect on survival in low-grade tumors (DFS: 74% vs. 75%, p = 0.98; OS: 81% vs. 84%, p = 0.93), it was associated with markedly improved outcomes in high-grade tumors for both DFS (80% vs. 58%, p < 0.05) and OS (71% vs. 57%, p < 0.05). This suggests that adenomyosis may exert a protective effect specifically against more aggressive tumors, potentially through modulation of the tumor microenvironment or alteration of hormonal pathways that influence high-grade tumor progression.

This differential effect may be explained by several mechanisms. High-grade tumors typically demonstrate a more aggressive biological behavior with less dependence on hormonal factors than low-grade tumors. The presence of adenomyosis, with its associated chronic inflammation, altered immune response, and modified cytokine profile, might create a microenvironment that counters the aggressive behavior of high-grade tumors. Specifically, inflammatory processes in adenomyosis may enhance immune surveillance, potentially leading to better tumor control in high-grade malignancies that would otherwise progress rapidly. Additionally, patients with adenomyosis often undergo gynecological examinations more frequently, potentially leading to earlier detection of malignancies, particularly those with aggressive features.

The lack of impact on low-grade tumors might be explained by their already favorable prognosis and slower growth patterns, making any additional protective effect of adenomyosis less detectable. This grade-dependent effect underscores the complex interplay between benign gynecological conditions and cancer biology, suggesting that the effect of adenomyosis is not uniform across all ovarian cancer subtypes and grades.

This study has several limitations. This retrospective design carries the risk of selection and information bias. However, the accuracy is strengthened by confirming adenomyosis through histopathological examination rather than through clinical diagnosis. As this was a single-center study, the findings may not be broadly generalizable. The sample size, although adequate, limited subgroup analyses, potentially underestimated the effects. A post-hoc power analysis (80% power, HR = 2.0) suggested that smaller effects may have gone undetected.

Our study highlights that ovarian cancer patients with adenomyosis tend to present with earlier stage disease, lower-grade tumors, and smaller tumor sizes, with a particular association with the endometrioid histological subtype. Furthermore, adenomyosis appeared to confer a survival advantage specifically in patients with high-grade tumors, although it was not an independent predictor of survival outcomes in the overall cohort. Clinically, these findings suggest that adenomyosis could potentially influence the disease presentation and biology in ovarian cancer, offering a basis for stratifying patients during diagnosis and management.

Future therapeutic strategies should explore the modulation of hormonal pathways and inflammatory processes common to both adenomyosis and ovarian cancer, potentially affecting disease progression. The strong association between adenomyosis and endometrioid subtype suggests that targeting shared pathogenetic mechanisms might yield therapeutic benefits, particularly for this histological variant. Additionally, identifying specific molecular markers related to adenomyosis in patients with ovarian cancer could guide personalized treatment approaches, particularly for patients with high-grade tumors, where adenomyosis appears to exert its most significant protective effect. Further multicenter prospective studies with larger sample sizes are warranted to validate these findings and determine whether adenomyosis-targeted therapies could improve ovarian cancer outcomes.

In conclusion, this analysis of 226 ovarian cancer patients demonstrated that adenomyosis was associated with favorable clinicopathological features, including earlier stage disease, lower-grade tumors, smaller tumor sizes, and endometrioid histology. Although adenomyosis improved disease-free survival, particularly in high-grade tumors, it was not an independent prognostic factor. Our findings suggest that adenomyosis may influence ovarian cancer biology through shared hormonal and molecular pathways, with implications for personalized treatment. These results provide a foundation for future research on the relationship between adenomyosis and ovarian cancer, which could inform therapeutic strategies.

## What does this study add to the clinical work?

Adenomyosis in ovarian cancer is associated with more favorable clinicopathological features, including earlier-stage disease, lower-grade tumors, and a strong correlation with endometrioid histology (OR = 2.89), while negatively associated with serous carcinoma (OR = 0.39). Although adenomyosis does not affect survival in low-grade tumors, it significantly improves disease-free (80% vs. 58%) and overall survival (71% vs. 57%) in high-grade tumors, suggesting a potential role in personalized treatment.

## Data Availability

Data supporting the findings are available from the corresponding author upon request.
